# The Relationship between Cortisol Activity during Cognitive Task and Posttraumatic Stress Symptom Clusters

**DOI:** 10.1371/journal.pone.0144315

**Published:** 2015-12-02

**Authors:** Hongxia Duan, Li Wang, Liang Zhang, Jing Liu, Kan Zhang, Jianhui Wu

**Affiliations:** 1 Key Laboratory of Behavioral Science, Institute of Psychology, Chinese Academy of Sciences, Beijing, China; 2 University of Chinese Academy of Sciences, Beijing, China; 3 Key Laboratory of Mental Health, Institute of Psychology, Chinese Academy of Sciences, Beijing, China; University of Toledo, UNITED STATES

## Abstract

**Background:**

The latest development in the dimensional structure of posttraumatic stress disorder (PTSD) is a novel 6-factor model, which builds on the newly released DSM-5. One notable gap in the literature is that little is known about how distinct symptom clusters of PTSD are related to hypothalamic–pituitary–adrenal (HPA) axis activity when people perform a relatively less stressful cognitive task. The purpose of this study was to investigate the relationship between cortisol activity when individuals perform cognitive tasks in the laboratory and a contemporary phenotypic model of posttraumatic stress symptomatology in earthquake survivors.

**Methods:**

Salivary cortisol while performing cognitive tasks was collected and analyzed in 89 adult earthquake survivors. The PTSD Checklist for the DSM-5 (PCL-5) was used to assess the severity of total PTSD as well as six distinct symptom clusters. Regression analyses were conducted to examine the associations between the six distinct PTSD symptom clusters and cortisol profiles.

**Results:**

The results showed that the score of the negative affect symptom cluster, but not anhedonia or other clusters, was positively associated with cortisol levels before and during the cognitive tasks.

**Conclusion:**

The results showed that higher cortisol levels before and during cognitive tasks might be specifically linked to a distinct symptom cluster of PTSD—negative affect symptomatology. This suggests that a distinction should be made between negative affect and anhedonia symptom clusters, as the 6-factor model proposed.

## Introduction

Exposure to a major disaster has been linked to psychological disorders, including an increase in the incidence of posttraumatic stress disorder (PTSD). According to an epidemiological study with a large sample size, the prevalence of PTSD among adult survivors of the Wenchuan earthquake was 15.57% [1]. PTSD is a clinical syndrome with high heterogeneity, and is composed of distinct symptom clusters. The different symptoms in these clusters are used in various combinations to define PTSD status, and the mechanisms underlying the distinct symptom clusters are also disparate [2]. Researchers have demonstrated that the distinct symptom clusters of PTSD exhibit potential correlations with neurobiological processes. For example, norepinephrine transporter availability in the locus coeruleus was positively associated with anxious arousal rather than dysphoric arousal symptoms [3], and 5-HT receptor density in the hippocampus was negatively associated with numbing symptoms [4].

Evidence of abnormal hypothalamic–pituitary–adrenal (HPA) activity in many psychiatric disorders, including PTSD, has long suggested HPA hormones to be biomarkers of disease or treatment response [5]. Research that explores cortisol response to stress could provide further information about the function of the HPA axis in PTSD. To our knowledge, there have been only a few such studies, and the results were inconsistent. Elzinga et al. [6] examined the cortisol response to a specific stressor (personalized traumatic script) in abused women with and without PTSD. They found higher salivary cortisol levels before, during, and after the stressor in the PTSD group than in the non-PTSD group. Meanwhile, cortisol release before and after exposure to the stressor was positively correlated with total PTSD severity. In Bremner et al.’s [7] study, civilians with PTSD related to childhood abuse displayed increased cortisol levels before and during a stressful cognitive challenge (challenging arithmetic task, Stroop task, problem solving, etc., under time pressure and with negative feedback) compared with healthy controls. However, another two studies with a combat sounds stressor [8] and Trier Social Stress Test (TSST, a standardized psychosocial stress test in the laboratory) [9] found no difference in cortisol levels between PTSD and healthy control groups.

Understanding how cortisol correlates with PTSD symptom clusters might offer biological indexes about distinct aspects of PTSD symptoms and improve future treatments. Recent studies have begun to explore the association between distinct PTSD symptom clusters and the cortisol response to stress. Elzinga et al. [6] found that cortisol release before and after exposure to the trauma scripts was positively correlated with re-experiencing and hyperarousal symptoms, but not with avoidance. Using a sample of high-exposure 9/11 survivors, Dekel et al. [10] found that elevated cortisol levels after trauma recollections were associated with the severity of re-experiencing and avoidance symptom clusters. Some longitudinal studies have also attempted to explore the relationship between specific symptom clusters from the 3-factor model and cortisol levels. Hawk et al. [11] found that greater emotional numbing predicted a lower urinary cortisol level six months after a motor vehicle accident. With the same trauma type, Delahanty et al. [12] revealed a negative association between cortisol level at the time of the accident and intrusive/avoidant thoughts 1 month later. Although these studies demonstrated a specific association between abnormal HPA-axis reactivity and the phenotypic heterogeneity of PTSD symptomatology, \they all used the 3-factor DSM model, which has received limited empirical support. Recently, using the four symptom clusters identified in the DSM-5 published in May, 2013 [13], Stoppelbein and Greening [14] have found higher cortisol levels to be associated with greater severity of numbing symptoms among mothers of children diagnosed with cancer. The latest development in this literature is a novel 6-factor model, which was derived from confirmatory factor analysis (CFA) in an epidemiological sample of Chinese earthquake survivors, and builds on the newly released DSM-5 [13]. The 6-factor model is comprised of intrusion (B1–B5), avoidance (C1–C2), negative affect (D1–D4), anhedonia (D5–D7), dysphoric arousal (E1–E2, E5–E6), and anxious arousal (E3–E4) and emerged as the best-fitting among the other five DSM-5 models [15]. The main difference between this CFA-derived 6-factor model and other models is that a single symptom cluster of “negative alterations in mood and cognitions” in the DSM-5 model was divided into a negative affect cluster and an anhedonia cluster. Previous theoretical studies suggested that negative affect enhancement and positive affect deterioration are distinct constructs in mood and anxiety disorders [16–18]. Evidence from empirical studies also shows a differentiation between negative and positive affect. For example, Smyth et al. [19] explored the relationship of both momentary positive and negative affect with salivary cortisol levels, and found negative affect to be associated with higher cortisol levels and positive affect to be associated with lower cortisol levels. With the same method, other researchers found a positive association between negative affect and cortisol levels, but no significant relationship between positive affect and cortisol levels [20, 21]. Using a standardized laboratory stressor (TSST), results from two studies showed that negative affect increase, rather than positive affect deterioration, induced by psychosocial stress was positively associated with cortisol response levels [22, 23]. Because all these results were from healthy individuals, how cortisol correlates of negative affect and anhedonia (positive affect deterioration) from the new 6-factor model of PTSD is still unknown.

The aim of the current study was to explore the correlation between the cortisol release profile under less stressful cognitive tasks without trauma-related stimuli and the newly proposed phenotypic 6-factor model of PTSD symptomatology in a sample of Chinese earthquake survivors. Previous studies have all involved the cortisol response of PTSD patients to stressful challenges; however, in everyday life we may encounter more neutral events than stressful ones. It is very likely that the individuals with posttraumatic stress symptoms experience more stress even under relatively neutral situations in daily life. Several taxometric investigations have suggested a dimensional model, rather than a distinct categorical model, to be representative of PTSD [24–26], which means that PTSD may be better featured as the upper extremity of a posttraumatic stress continuum rather than a discrete mental disorder (individuals with PTSD vs. those without PTSD). The approach of using homogeneous symptom clusters for mental disorders is consistent with the Research Domain Criteria (RDoC) project initiated by the National Institutes of Mental Health (NIMH) [27]. Furthermore, it would be more informative to have a full-range analysis of symptom severity, because this kind of analysis could result in greater statistical power and less parameter estimation bias [28]. Thus, all participants, not just probable PTSD cases, were included in the final analysis. Based on the evidence from healthy individuals, we hypothesized that in the current PTSD symptomology study, there might be a distinction between negative affect and anhedonia, that is, negative affect symptoms might be positively associated with cortisol levels, while anhedonia symptoms might be unrelated to cortisol levels when performing the cognitive tasks.

## Methods

### Participants

This study was part of a large project addressing the relationship between PTSD symptomology and biology/cognition. Participants were recruited from one of the largest rebuilt communities located in Hanwang Town, which was almost completely destroyed by the 2008 Wenchuan earthquake. Sampling procedures were as follows: (1) the sample unit was based on the household and only one member in each household was randomly selected to take part in the project; (2) eligible participants were those older than 16 years, who had experienced the earthquake personally; (3) participants with mental retardation or major psychiatric disorder (e.g., schizophrenia and organic mental disorders) were not included; (4) among the eligible members in each household, the individual whose birthday was closest to the investigation date was preferred, and if this individual was unavailable, the family member with the next closest birthday was selected [15]. All the procedures were completed by investigators who were trained psychiatrists, clinical psychologists, psychotherapists, or graduate psychology students.

The participants should spend one day in the laboratory to complete the procedure during the period 13–31 December, 2013, approximately five and a half years after the earthquake. Because of the potential impact on the HPA axis, the following exclusion criteria were employed: endocrine, neurological or psychiatric medicine in recent months; current alcohol (more than two alcoholic drinks daily) or nicotine (more than two packs of cigarettes/day) abuse; current periodontitis or oral wound; long-term overnight shift work or irregular circadian rhythm; mental retardation or any history of major psychosis (e.g., schizophrenia and organic mental disorder); any history of serious head trauma; current acute inflammation or allergy. About one-quarter of the study participants were taking medicines; the most commonly reported medicine was health-care related.

In total, 89 survivors were recruited. They gave written informed consent and were paid for their participation. Seven individuals were excluded because two or three of their cortisol samples were missing, leaving a final sample of 82 participants (age range: 21–65 years). There were nine participants with a single missing cortisol sample, and the missing data were interpolated [7]. Demographic variables are shown in Table 1. The experiment was approved by the Ethics Committee of Human Experimentation in the Institute of Psychology, University of Chinese Academy of Sciences. Participants under 18 provided written informed consent from their next of kin, caretakers, or guardians on their behalf.

**Table 1:** Demographic variables of the study group.

Variable	n	%	Mean	SD
***Sex***				
**Male**	37	45.10		
**Female**	45	54.90		
***Age (yrs)***			48.12	10.28
***Educational level***				
**High school or above**	33	40.20		
**Less than high school**	49	59.80		
***Marital status***				
**Married**	70	85.40		
**Single/divorced/separated widowed**	12	14.60		
***Sleep duration during the night previous night (h)***			7.24	1.69
***Drinking (mL/d)***	13	15.90	123.08	83.83
***Smoking (no*.*/d)***	26	31.70	15.83	8.07
***State anxiety***			34.66	8.33

### Procedure

Demographic data (gender/age/educational level/marital status), PTSD Checklist for DSM-5 (PCL-5), the Center for Epidemiological Studies Depression Scale (CESD), and the Trauma Exposure Scale were completed in the participants’ own community, under the instruction of investigators, before they came to the laboratory. They were told to obtain their usual amount of rest the night before the experiment, and asked to have a light breakfast and to refrain from drinking and smoking until after the procedure. On arrival at the laboratory, participants were instructed to rest for half an hour while basic information (drinking habits/smoking habits/sleep duration during the previous night) was gathered and the state anxiety level was measured. Drinking habits were assessed by asking participants whether they drank alcohol, and how many units they drank each day. Smoking habits were assessed by asking whether participants smoked and how many cigarettes they consumed each day. Participants were informed of the experimental procedure. All participants were unfamiliar with the experiment. The cognitive tests applied in our experiment were based on disrupted prefrontal function in the PTSD domain [29, 30], and included the modified Stroop task, anticipation task, and Go/NoGo task. The performance of these tasks is beyond the scope of this paper and will be reported elsewhere.

To avoid contamination of saliva, participants were asked not to do strenuous physical exercise, smoke, drink, or eat after they arrived at the laboratory until saliva sampling had been completed. After half an hour’s rest, the first salivary cortisol sample (S1) from each participant was obtained. The participants completed two cognitive tasks; the Stroop task of identifying the color overlapping a picture of a face while ignoring the emotion in the facial expression, and the anticipation task, which involved pressing a button when the stimulus on the screen disappeared. These tasks took about half an hour. The second salivary cortisol sample (S2) was then collected, followed by the last cognitive task (the Go/NoGo task, which involves responding to one of three colors in a picture frame while withholding response to the other two colors) for about half an hour. The third salivary cortisol sample (S3) was then collected. None of the stimuli used in the study was associated with trauma experience, and there was no feedback on the results.

### Questionnaires

The PTSD symptoms were assessed by the PCL-5 [31, 32]. The PCL-5 is a 20-item self-report scale, which was adapted from the original PCL to map onto the PTSD symptoms of the newly released DSM-5. Individuals were requested to rate each item on a 5-point scale (0 = not at all, 4 = extremely) to measure the severity of a specific symptom during the past month. The original PCL was found to have solid reliability and validity [33, 34]. The version used here was translated into Chinese for use in trauma-related research [35, 36]. In the current study, participants were instructed to complete the PCL in relation to the Wenchuan earthquake.

Participants were also instructed to complete the CESD, which is a 20-item scale for depressive symptoms [37]. Respondents indicated the extent of the symptoms they had experienced in the past week on a 4-point scale (0 = rarely or none of the time, 3 = most or all of the time). The version used here was translated into Chinese and has demonstrated good reliability and validity [38].

The intensity of each participant’s trauma exposure was assessed by asking participants to respond with “yes” or “no” (yes = 1, no = 0) to the following questions: (1) Were you trapped under rubble? (2) Were you injured? (3) Were you disabled by injuries? (4) Did you participate in rescue efforts? (5) Did you witness the death of someone? (6) Did you see mutilated bodies? (7) Did any family members die in the disaster? (8) Were any family members injured? (9) Did any friend or neighbor die in the disaster? (10) Did you lose your livelihood as a result of the disaster? The intensity of trauma exposure was calculated by adding the score for each item [15].

The state anxiety level before the experiment was assessed by the State-Trait Anxiety Inventory (STAI) [39], which is one of the universal scales used to measure anxiety [40]. The version used here was translated into Chinese and has demonstrated good reliability and validity [41, 42].

### Cortisol measurement

Saliva samples were collected by asking participants to chew lightly on a Salivette swab for 2 min [43]. The samples were kept frozen (–20°C) until assay. The first sample was collected between 09:50 and 15:21; 22 (26.8%) provided their first saliva sample between 09:50 and 10:19, 38 (46.4%) between 12:43 and 13:54, and 22 (26.8%) between 14:06 and 15:21. The mean (SD; range) times between sample collections were 39.00 min (12.54; 18.00–81.00) between samples 1 and 2, and 68.98 min (12.42; 49.80–111.00) between samples 1 and 3.

Samples were thawed and centrifuged at 3200 rpm for 10 min. Cortisol concentration was analyzed using electrochemiluminescence immunoassay (Cobas e 601, Roche Diagnostics, Numbrecht, Germany), with sensitivity of 0.500 nmol/L (lower limit) and a standard range in assay of 0.5–1750 nmol/L. Intra- and interassay variations were below 10%.

### Data analysis

Data distribution of all variables was examined by the Shapiro–Wilk test. Logarithmic-base-10 transformations were applied to non-normally distributed variables (e.g., cortisol values). Demographic descriptive statistics were computed for sample characteristics (gender, age, marital status, and educational level), sleep duration during the last night, and drinking and smoking habits. Preclinical variables for depression level, trauma exposure, and PTSD severity, including six symptom clusters, were also computed.

Except for the saliva concentration at three single time points, mean level and area under the curve with reference to the ground (AUC_g_) were computed [44]. Cortisol levels were transformed using logarithmic-base-10 transformations before analysis, while raw data were reported to allow comparison with other studies. Repeated measures analysis of variance (ANOVA) was carried out on the three cortisol samples. The Greenhouse–Geisser correction for degrees of freedom was applied when the sphericity assumption was violated. Where repeated measures ANOVA procedures revealed a significant main effect, *post hoc* analyses of least square difference were used to examine the specific effects and significant levels.

Bivariate associations between the severity of total PTSD and distinct symptom clusters and cortisol metrics were evaluated with Pearson correlation analyses. Multivariate regression analyses were conducted to examine the associations between total PTSD severity and each distinct PTSD symptom and cortisol metrics; the total PTSD score or symptom cluster scores of PTSD that were associated with cortisol levels at the *p* < 0.05 level in bivariate analyses were treated as predictors and each of the cortisol values were treated as dependent variables [4, 45]. Meanwhile, when demographic variables, drinking/smoking habit, depression, and trauma exposure were associated with cortisol levels at the *p* < 0.05 level in bivariate analyses, they were also entered as independent variables in the multivariate regression. To evaluate whether the sampling time of day influenced associations between the cortisol metrics and PTSD severity and symptom clusters, sampling time of day was divided into late morning to early afternoon, and mid-afternoon to late afternoon, in accordance with Lee et al.’s [46] study. When this variable was associated with cortisol at the *p* < 0.05 level, it was added to the regression analysis. All *p* values below 0.05 were considered statistically significant.

## Results

The demographic data are presented in Table 1. Table 2 shows preclinical variables (means and standard deviations [SDs]) for PCL-5 as well as symptom clusters, CESD (depression), and trauma exposure scores. The cortisol metrics under the cognitive tasks are illustrated in Table 3. The repeated measures analysis comparing the three samples revealed a significant time main effect (*F*(2, 81) = 6.18, *p* < 0.01). *Post hoc* analyses showed that cortisol levels in sample 3 (S3) and sample 2 (S2) were significantly higher than in sample 1 (S1; *p*_*s*_ < 0.05), and cortisol levels in S3 were marginally higher than those in S2 (*p* = 0.08).

**Table 2:** Descriptive statistics of PCL-5 and symptom clusters, CESD, and trauma exposure scores.

	PCL-5 (Total)	IN	AV	NA	AN	DA	AA	CESD	TE
**Mean**	28.84	9.33	3.49	4.40	3.10	5.30	3.23	39.80	4.82
**SD**	17.0	4.70	2.36	3.96	2.79	3.89	2.16	9.89	1.63
**Range**	1–69	1–20	0–8	0–14	0–11	0–16	0–8	24–67	1–8

PCL-5 = PTSD Checklist for DSM-5; IN = intrusion; AV = avoidance; NA = negative affect; AN = anhedonia; DA = dysphoric arousal; AA = anxious arousal; SD = standard deviation; CESD = Center for Epidemiological Studies Depression Scale; TE = trauma exposure.

**Table 3:** Cortisol levels at three time points (S1/S2/S3) during the cognitive tasks (nmol/L).

	S1	S2	S3	Mean	AUC_g_
**Mean**	11.13	11.62	12.77	11.84	13.26
**SD**	5.63	5.19	7.77	5.03	5.61

S1 = cortisol level before the cognitive tasks; S2 = cortisol level during the cognitive tasks; S3 = cortisol level after the cognitive tasks; Mean = mean value of the three cortisol samples; AUC_g_ = area under the curve with reference to the ground; SD = standard deviation.

### Tests of association between cortisol and PTSD symptom clusters

**Fig 1:**
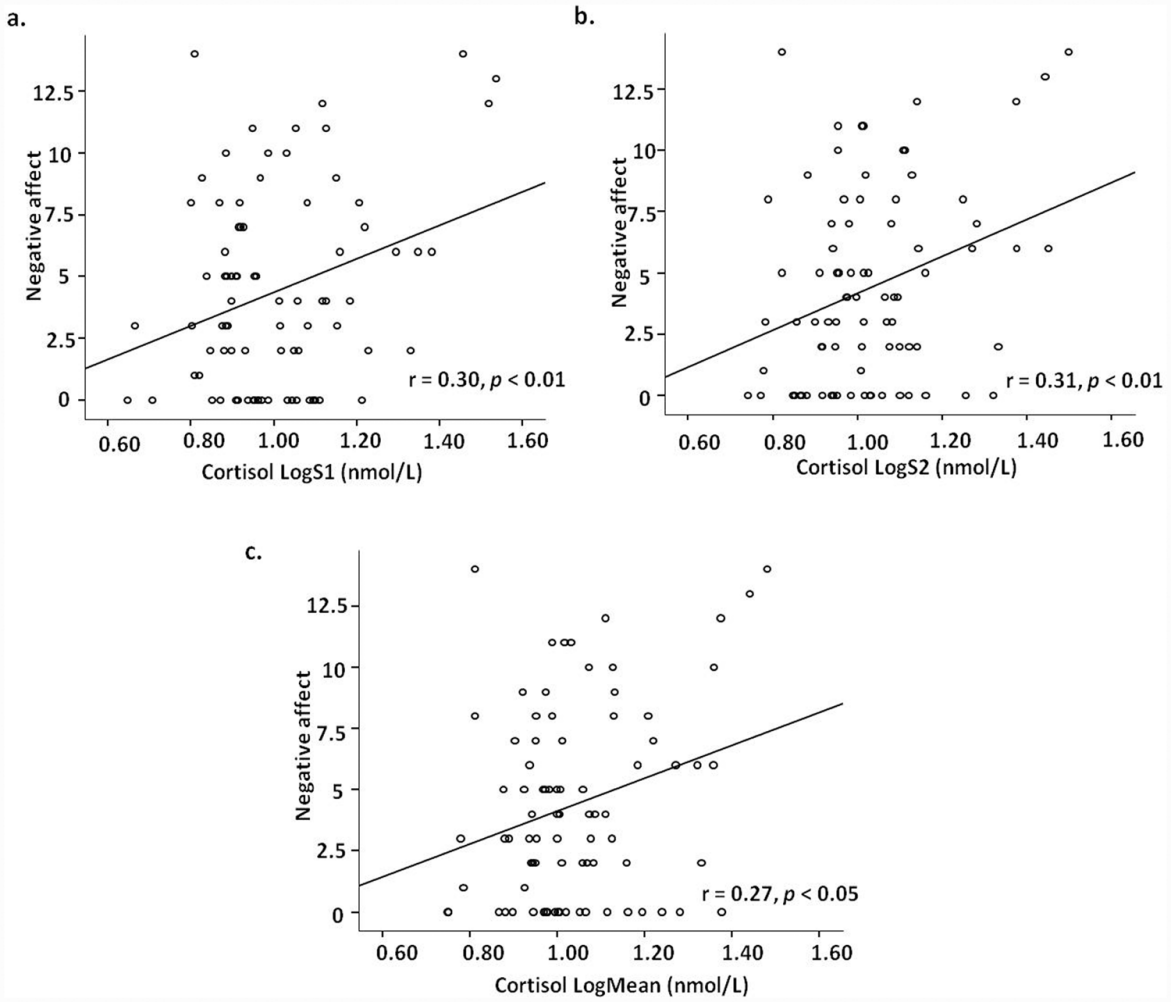
Scatter plot of correlation between negative affect symptom and (a) the log-transformed first cortisol sample (LogS1); (b) the second cortisol sample (LogS2); and (c) the mean cortisol sample during the cognitive tasks.

Bivariate correlations showed that sampling time was significantly negatively correlated with cortisol level in S1, prior to the cognitive tasks (r = –0.37, *p* < 0.01). None of the demographic, state anxiety, trauma-related exposure, or depression variables was associated with S1 (|r|_s_ < 0.21, *p*_s_ > 0.05). Although total PCL-5 scores were not significantly associated with S1 (r = 0.16, *p* = 0.16), the negative affect subscale scores from the 6-factor model of PTSD were significantly associated with S1 (r = 0.30, *p* < 0.01) (see Fig 1a). Thus, in the multivariate regression analysis, sampling time and negative affect score were input as independent variables. Results (adjusted R^2^ = 0.18) showed that the severity of negative affect was significantly positively associated with S1 (β = 0.26, t = 2.55, *p* < 0.05, see Table 4). The sampling time of S1 was also negatively correlated with cortisol level (β = –0.33, *p* < 0.01).

**Table 4:** Relationship between cortisol metrics and the negative affect symptom clusters.

Cortisol	r	B	β	t	*p*
**S1**	0.30	0.01	0.26	2.55	0.01
**S2**	0.31	0.01	0.24	2.10	0.04
**Mean**	0.27	0.01	0.23	2.18	0.03

S1 = cortisol level before the cognitive tasks; S2 = cortisol level during the cognitive tasks; Mean = mean value of the three cortisol samples.

Correlation analysis indicated that cortisol level during the cognitive tasks (S2) was not associated with total PCL-5 score (r = 0.19, *p* < 0.05), but was significantly correlated with the demographic variables of age (r = 0.24, *p* < 0.05), drinking (r = 0.24, *p* < 0.05), and negative affect subscale scores (r = 0.31, *p* < 0.01; see Fig 1b). The multivariate regression result (adjusted R^2^ = 0.12) showed that severity of negative affect symptoms was significantly positively associated with this cortisol sample (β = 0.24, t = 2.10, *p* < 0.05, see Table 4), while age and drinking were not associated with cortisol level (*p* > 0.05).

For cortisol level after the cognitive tasks (S3), there were no significant correlations between independent and dependent variables (*p*_*s*_ > 0.05).

Correlation results revealed that the mean cortisol level was significantly associated with sampling time (r = –0.29, *p* < 0.01) and severity of negative affect symptoms (r = 0.27, *p* < 0.01) (see Fig 1c), but not with total PCL-5 score (r = 0.13, *p* > 0.05). The multivariate regression analysis (adjusted R^2^ = 0.11) revealed that severity of negative affect (β = 0.23, t = 2.18, *p* < 0.05, see Table 4) was correlated with mean cortisol levels. The sampling time was also associated with mean cortisol levels (β = –0.26, t = –2.45, *p* < 0.05).

Only sampling time and age were significantly correlated with AUC_g_, (r = 0.31 and 0.31, respectively, *p* < 0.05).

## Discussion

This study investigated the relationship between cortisol levels under less stressful cognitive tasks and the severity of distinct symptom clusters of PTSD from the 6-factor model based on the DSM-5. The main finding was that only the negative affect symptom cluster, but not total PTSD severity or other clusters, was associated with cortisol levels.

The PTSD phenotype was composed of distinct symptom dimensionality, which might be linked with different psychopathological and physiological processes [4, 47]. With the aid of more homogeneous symptom clusters in the PTSD field, the chance of identifying distinct contributions from specific symptom dimensions to biological processes can be improved and the heterogeneity of results can be reduced. Following this approach, linking the cortisol level with a contemporary 6-factor model provides more sophisticated information about the role of cortisol in relation to the distinct symptom clusters that compose the PTSD phenotype. The results in our study revealed a specific association between cortisol levels and negative affect symptoms. Specifically, the first cortisol sample (S1) was positively associated with the negative affect cluster. S1 was collected immediately before the cognitive task, and the level of cortisol in S1 may reflect the HPA activity during the period of anticipation prior to the upcoming task [7, 48]. Thus, the positive relationship between S1 and the negative affect cluster might suggest that higher negative affect cluster scores predict increased anticipation-related HPA activity. The negative affect cluster was also positively associated with the second cortisol sample (S2), which was collected during the cognitive tasks, and might suggest that the negative affect cluster also predicts an increase in cortisol activity during the cognitive task itself.

The cortisol level immediately after the completion of tasks (S3) was the highest of the three samples, but showed no significant association with negative affect or other clusters in our study. This result may suggest that with further task execution, the links between negative affect and cortisol activity may disappear and a coping process may instigate the dynamic change in the relationship between negative affect and cortisol activity [49, 50].

However, anhedonia was not significantly associated with cortisol levels in the current study. This was consistent with other studies that used the momentary assessment method [20, 21, 51]. In Lovallo et al.’s study [52], participants were required to use active and passive coping strategies when exposed to aversive stimuli (noise and electric shock). They found that cortisol increased equally with both coping strategies, and suggested that the negatively affective nature of the tasks rather than the positive affect enhancement was related to cortisol. Meanwhile, Lovallo et al. [53] compared the cortisol levels under aversive conditions with cortisol under appetitive conditions, and found that cortisol increased significantly during the aversive task but remained unchanged during the appetitive task. Thus, these previous results suggest that cortisol response was representative of the negative affective nature. Our results from the PTSD population extend this assumption.

Our results were very consistent with the literature about the relationship between cortisol level and negative/positive affect. Many empirical studies have demonstrated a positive association between negative affect assessed by daily reports and diurnal cortisol level [19–21, 54]. However, the relationship between positive affect (assessed by daily report) and cortisol level was more equivocal, with negative associations [19] or no significant associations [20, 21, 51]. Furthermore, results from the stress induction procedure in the laboratory showed that the increase in negative affect was accompanied by higher levels of cortisol after stress, yet decreases in positive affect were not associated with cortisol levels [22, 23]. The association between negative affect and alterations in cortisol reactivity has also been well documented in depressed patients [55]. The current study revealed for the first time that cortisol level during cognitive tasks was associated with a negative affect symptom cluster rather than anhedonia in the PTSD population.

In the DSM-5 model, PTSD negative affect and anhedonia were grouped into a single symptom cluster as negative alterations in mood and cognitions. However, the cluster of negative alterations in mood and cognitions is a diverse construct within PTSD, which consists of symptoms involving enhanced negative affect/general distress and symptoms of reduced positive affect/anhedonia. Previous theoretical and empirical studies also suggest that negative affect enhancement and positive affect deterioration are distinct constructs in mood and anxiety disorders [16–18]. Meanwhile, according to the RDoC project, negative valence and positive valence have also been specified as two domains in the psychopathology field [56, 57]. This study showed that only the negative affect cluster, and not the anhedonia cluster, was associated with cortisol activity, which provides neuroendocrine support for the current distinction of negative affect and anhedonia PTSD symptoms in the 6-factor model.

Notably, total PTSD severity was not associated with any cortisol metrics in our research. This result is in line with the observation of cortisol response to a stressful cognitive challenge in a childhood abuse-related PTSD population [7]. However, Elzinga et al. [6] found that severity of posttraumatic symptoms was positively associated with cortisol levels in a group of abuse-related PTSD females who were exposed to trauma scripts. The inconsistent results might be caused by the stress experienced during the assessment [58]. While Elzinga et al. [6] used trauma scripts in their study, trauma-free cognitive tasks were employed to induce cortisol secretion in both Bremner et al.’s [7] and our study. Demographic factors, such as gender, might also have influenced the outcome. In the study by Elzinga et al., only females were recruited, while both males and females were included in Bremner et al.’s study and our study.

There are some limitations to this study. First, the cross-sectional design limited our ability to determine causality regarding the observed association between cortisol level and PTSD symptom severity. Second, participants in our study were scheduled to participate at different times of the day, thus the cortisol sampling time varied. Diurnal rhythm in cortisol levels and HPA-axis sensitivity might have affected the results in our study. However, Kudielka et al. [59] assessed the HPA-axis response to psychosocial challenges based on five independent studies conducted in the same laboratory and found that comparable HPA-axis stress responses can be measured in the morning and afternoon. Furthermore, the results remained consistent after the sampling time was controlled in our multivariate regression analysis. Finally, the present study did not differentiate between the PTSD and non-PTSD groups, and therefore the results only apply to the traumatized population.

In conclusion, our research on the clinical–neuroendocrine association suggests that negative affect symptoms, but not anhedonia or other clusters, are associated with increased cortisol levels before and during common cognitive tasks. This suggests that a distinction should be made between negative affect and anhedonia symptom clusters, and provides empirical evidence for the 6-factor model.
